# Economics of open tibial fractures: the pivotal role of length-of-stay and infection

**DOI:** 10.1186/s13561-017-0168-0

**Published:** 2017-09-25

**Authors:** Harm Hoekstra, Bart Smeets, Willem-Jan Metsemakers, Anne-Cécile Spitz, Stefaan Nijs

**Affiliations:** 1 Department of Trauma Surgery, University Hospitals Leuven, Herestraat 49, B-3000 Leuven, Belgium; 2 Department of Development and Regeneration, KU Leuven - University of Leuven, B-3000 Leuven, Belgium; 3 Dataroots, B-1040 Brussels, Belgium; 4 Faculty of Medicine, KU Leuven - University of Leuven, B-3000 Leuven, Belgium

**Keywords:** Costs analysis, Open tibial fractures, Healthcare financing

## Abstract

In order to define strategies to curb the continuing increase in healthcare costs, we describe the cost breakdown of open tibial fractures. Twenty-seven clinical and process variables were recorded retrospectively, and five main hospital related cost categories were defined. Three multivariate linear models were fitted to the data. Total healthcare costs of open tibial fractures were almost twice as high compared to closed fractures and mainly existed of hospitalization costs. Length-of-stay (LOS) was found to be the most important variable driving the healthcare costs of open tibial fractures. Deep infection lead to a 6-fold increase of LOS and 5-fold increase in total healthcare costs of open tibial fractures. Therefore, appropriate international consensus guidelines are required to improve not only the patient outcome (infection prevention) but also reduce overall healthcare cost by focusing on reducing the LOS.

## Background

Healthcare costs have increased significantly over the past decades. In Belgium, health expenditure currently accounts for approximately 10% of its GDP (gross domestic product), up from 8% in 2000 [1]. This puts Belgium on the top 10 list of OECD (Organization for Economic Co-operation and Development) countries with the highest healthcare expenditure, which is led by the United States with 16.4% [2]. The Belgian healthcare sector has been urged to find ways to keep the healthcare system viable. Therefore, multiple studies have focused on different facets of healthcare utilization and related costs. Recently, we reported an exploratory analysis of the healthcare costs associated with the treatment of AO/OTA (Arbeitsgemeinschaft fur Osteosynthesefragen/Orthopedic Trauma Association) type 44-B ankle fractures and defined strategies (e.g. immediate percutaneous intramedullary fibular fixation) to curb the continuing increase in healthcare costs [3]. Length-of-stay (LOS) was identified here as the main driver of the total healthcare costs.

In parallel, we want to understand the cost breakdown of open tibial fractures, since these fractures are associated with an increased complication rate (e.g. surgical site infection and nonunion) and treatment costs [4–6]. The distribution and treatment strategies of open tibial fractures vary considerably, due to its specific anatomic properties. Tibial fractures represent the most common open long-bone injuries with over 15% being open [7]. Nevertheless, we want to formulate strategies to reduce the cost of open tibial fractures.

Here we report a retrospective study of a large consecutive cohort of adult patients with either an AO/OTA type 41, 42 or 43 tibial fracture. We describe open tibial fractures in relation to patient demographics and operative characteristics. We define all hospital-related healthcare costs within the Belgium's healthcare system. Next, in order to define clear-cut clinical pathways and keep the healthcare system vital, we identify a subset of clinical variables that have a significant influence on costs and LOS, and determine whether these variables are actually related to the total healthcare costs. We hypothesized that Gustilo type 3 open tibial fracture patients will incur higher costs.

## Methods

### Patients

A total of 358 patients with an acute AO/OTA type 41, 42 and 43 tibial fracture were included in this study. All patients were treated at the Traumatology Department of University Hospitals Leuven between January 2009 and January 2014. Follow-up was until January 2016, allowing a maximum follow-up time of two years for all patients. Exclusion criteria were non-acute fractures (diagnosed more than 4 weeks after the accident), age < 18 years (*n*=29), other musculoskeletal trauma surgery during the follow-up (*n*=46), primary treatment elsewhere (*n*=23), , pathological fractures (*n*=1), and amputation (*n*=1) within 5 days after the accident. This study was conducted in compliance with national legislation and the guidelines of the ethics committee of University Hospitals Leuven. All tibial fractures related hospital stays and ambulatory consults of the selected patients were included in the analysis.

### Study variables

Twenty-seven variables were retrospectively recorded from an electronic database (KWS – UZ Leuven); 20 clinical variables (gender, age, ASA-score [American Society of Anesthesiologists physical status score], cardiovascular risk factors [CVRF], AO/OTA bone segment, AO/OTA fracture type, AO/OTA fracture group, open fractures, antibiotic therapy, delayed-staged surgery, type of definite surgery, superficial infection, deep infection, nonunion, other complications, debridement (i.e. necrotectomy), hardware removal, re-osteosynthesis, other operations, and mortality) and 7 process variables (LOS to definite surgery, total length of antibiotic therapy, total LOS, number of operations, number of hospital admissions, number of surgical day care admissions, number of ambulatory consults).

Comorbidity was recorded using the ASA (American Society of Anesthesiologists physical status) -score. The cardiovascular risk factors (CVRF) include: age, current cardiovascular diseases (e.g., cerebrovascular accident, myocardial infarction, peripheral artery disease), diabetes, rheumatoid arthritis, smoking, use of blood vessel narrowing drugs (e.g., beta blockers and ergotamine), dyslipidemia, hypercholesterolemia, and hypertension. All fractures were classified according to the AO/OTA classification system [8]; fractures were classified according bone segment (41 - proximal tibia, 42 - tibia shaft, 43 - distal tibia), fracture type (A - extra-articular, B - partial articular, C - complete articular) and fracture group (1 - simple, 2 - wedge, 3 - complex). Open fractures were subdivided by the Gustilo-Anderson classification (type 1-3), which was determined at the time of initial debridement in the operating room [9]. Antibiotic therapy was categorized as either prophylactic (maximum of 5 days) or therapeutic [5]. Delayed-staged surgery includes all patients that were treated according to a delayed surgery protocol (i.e., external fixator prior to definite surgery). The type of definite surgery was categorized as intramedullary fixation (e.g. nailing), plate osteosynthesis, screw osteosynthesis, external fixator or arthrodesis (joint fusion). Surgical site infection was either classified as superficial or deep infections, which were defined according to Dellinger et al. and CDC (center for disease control) -guidelines. A superficial wound infection was one located above the fascia, with erythema and tenderness. A deep infection was defined as an infection involving deeper tissues as muscular fascia and bone, which could necessitate removal of the implant [10, 11]. Nonunion was assessed using follow-up radiographs and defined according to the US Food and Drug Administration guidelines as a not completely healed fracture within 9 months of injury and without progression towards healing over the past 3 consecutive months [12]. Other tibial related complications were wound problems, screw loosening, hardware migration, loss of reduction, peroneal nerve injury, and joint contracture. Nearly all other tibial related operations were bone grafting, (free) muscle flaps, and knee arthroplasty. LOS to definite surgery was defined as the number of consecutive hospital admission days until definite surgery. Total LOS was defined as the number of consecutive hospital admission days during the stay for the definite treatment. Surgical day care admissions include all operations (i.e. removal of hardware) performed in outpatients who did not require hospital admission.

### Cost categories

Five main hospital related cost categories were defined: honoraria, materials, hospitalization (cost of daily patient care), daycare stay, and pharmaceuticals [3]. In summary: honoraria mainly consists of fees related to medical activities, mainly based on a fee-for-service principle (i.e. surgery, consults, and imaging). In the Belgium’s healthcare system, honoraria are independent of the rank of the surgeon as activities are billed under the attending physician. Material related costs refer to the actual implants and other required materials. To analyze the performance impact due to differences in LOS, the patient’s actual LOS was multiplied by the average national day based care fee (€410.84). The resulting sum was interpreted as the patient’s hospitalization related costs. Costs for daycare stay include all operations (i.e. removal of hardware) performed in the outpatients that do not require hospitalization. Pharmaceuticals costs are all costs for received drugs and blood products.

The costs described in this paper relate to the Belgium’s healthcare financing context and are exclusively hospital care-related. Furthermore, these costs comprise the majority of reimbursements paid to the hospital (by any party involved) in financing the care for a specific patient either directly or indirectly.

### Statistical methods

Continuous variables are presented as medians with interquartile distribution, whereas categorical variables are presented as numbers and percentages. Categorical variables were compared with the Pearson Chi-Square test. Comparison of continuous variables was performed using the Mann-Whitney *U* test.

In order to determine the variables that drive healthcare costs and LOS of open tibial fractures, three multivariate linear models were fitted to the data (in the form of *y* = *a* + *b*_1_ *x*_1_ +  ⋯  + *b*_*n*_ *x*_*n*_). Leave-one-out (LOO) cross validation was used to analyze generalizability 1. For all categorical variables, binary dummy variables were introduced to represent the categorical values. For each categorical variable, the number of dummy variables introduced equals the number of different values the categorical value can take minus one.

The dataset with binary dummy variables included has a rather high dimensionality in relation to the number of available observations. As fitting a linear regression model directly on this dataset would lead to an unstable model, the dimensionality of the dataset was first reduced using Lasso regression for each of the three models. The variables that received a non-negative coefficient after the Lasso fit were then used to fit a multivariate linear model. The choice for a linear model was made based on interpretability reasons.

Exploratory data analysis and subsequent building of the statistical model was done using R and the mlr framework [13, 14].

## Results

### Patient characteristics

Patient demographics, operative variables, and hospital stay-related variables are summarized in Table 1. Categorical variables are presented as numbers and percentages, and continuous variables as the median with interquartile distributions.

**Table 1:** Patient characteristics (*n* = 358)

Clinical characteristics	Gender	
	-Male	191 (53.4%)
	-Female	167 (46.60%)
	Age (years)	49 (36 – 62)
	ASA-score	
	-ASA 1	158 (44.1%)
	-ASA 2	158 (44.1%)
	-ASA 3	42 (11.7%)
	CVRF	198 (58.9%)
	AO/OTA bone segment	
	-41	122 (34.1%)
	-42	140 (39.1%)
	-43	96 (26.8%)
	AO/OTA fracture type	
	-A	119 (33.2%)
	-B	139 (38.8%)
	-C	100 (27.9%)
	AO/OTA fracture group	
	-1	162 (45.3%)
	-2	54 (15.1%)
	-3	142 (39.7%)
	Open fractures (Gustilo)	51 (14.2%)
	-1	26 (51.0%)
	-2	15 (29.4%)
	-3	10 (19.6%)
	Antibiotic therapy	
	-prophylactic	313 (87.4%)
	-therapeutic	45 (12.6%)
	Delayed-staged surgery	44 (12.3%)
	Type of definite surgery	
	-intramedullary nail	140 (39.1%)
	-plate osteosynthesis	144 (40.2%)
	-screw osteosynthesis	66 (18.4.1%)
	-external fixator	7 (2.0%)
	-arthrodesis	1 (0.3%)
	Superficial infection	7 (2.0%)
	Deep infection	12 (3.4%)
	Nonunion	18 (5.0%)
	Other complications	8 (2.2%)
	Debridement	19 (5.3%)
	Hardware removal	121 (33.8%)
	Re-osteosynthesis	14 (3.9%)
	Other operations	22 (6.1%)
	Mortality	11 (3.1%)
Process characteristics	LOS to definite surgery (days)	1 (0 – 3)
	Total length of antibiotic therapy (days)	1 (1 – 3)
	Total LOS (days)	7 (5 – 13)
	Number of operations	1 (1 – 2)
	Number of hospital admissions	1 (1 – 2)
	Number of surgical day care admissions	0
	Number of ambulatory consults	5 (3 – 8)

Categorical variables are presented as numbers and percentages, continuous variables as median with interquartile distributions

*Abbreviations: ASA-score* American Society, of Anesthesiologists physical status score, *CVRF* cardiovascular risk factors, *AO/OTA* Arbeitsgemeinschaft für Osteosynthesefragen/Orthopaedic Trauma Association, *LOS* length-of-stay

### Healthcare costs

The distribution of the 5 main cost categories (honoraria, materials, hospitalization, daycare stay, and pharmaceuticals) and total costs are summarized in Table 2. The costs are presented as the median and interquartile distributions.

**Table 2:** Healthcare costs (n=358)

Category	Per patient	Total	Relative share
Honoraria	€1,596 (1,182 – 2,556)	€775,515	21 %
Materials (implants & screws)	€1,055 (544 – 1,528)	€446,313	12 %
Hospitalization	€2,876 (1,951 – 5,341)	€1,901,368	51 %
Daycare stay	€0 (0 – 86)	€36,931	1 %
Pharmaceuticals	€1,029 (870 – 1,789)	€553,071	15 %
Total	€6,962 (4,932 – 10,972)	€3,713,198	100%

The per patient costs show the median followed by interquartile range

### Descriptive statistics of open tibial fractures

**Fig. 1:**
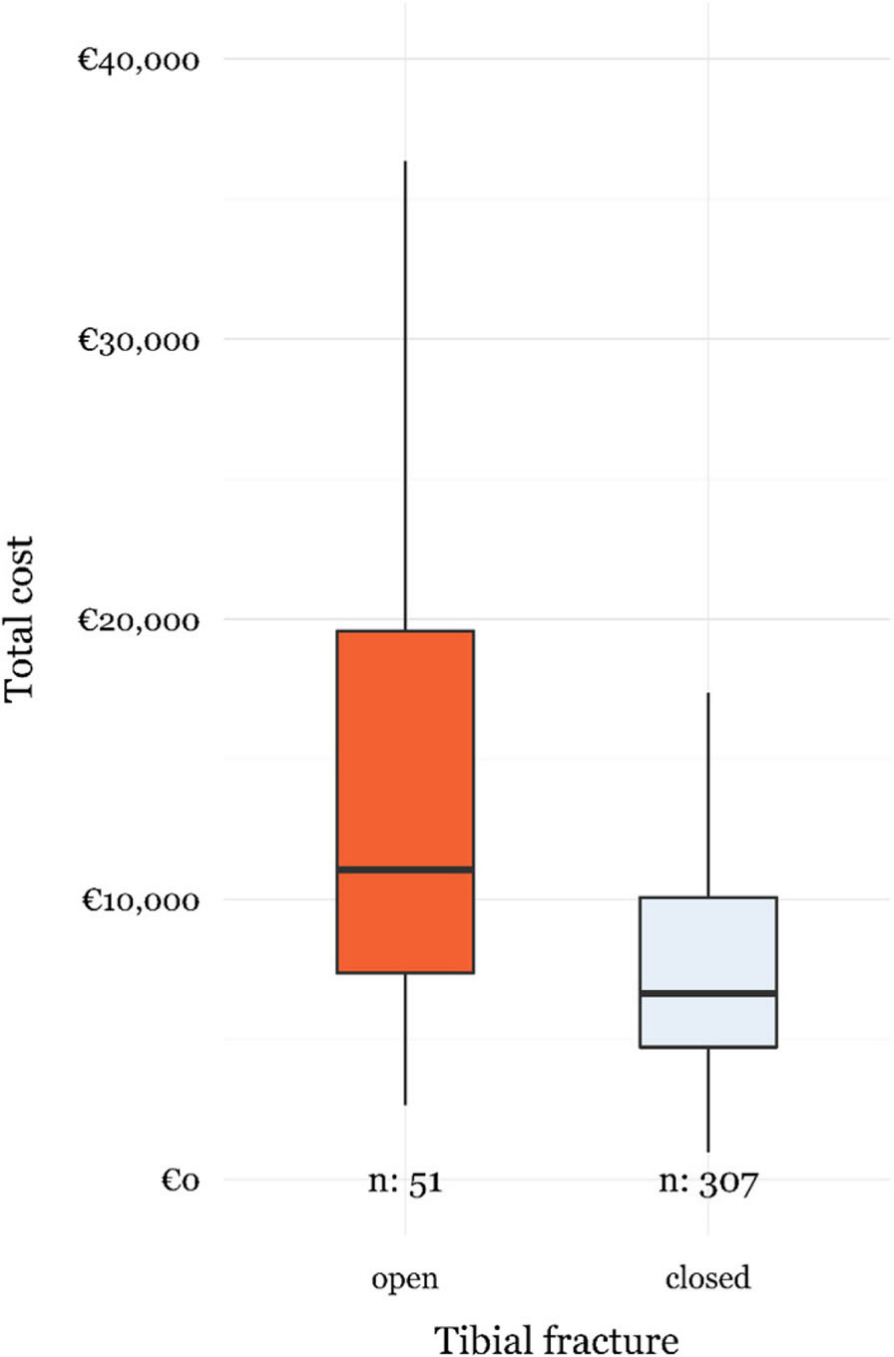
Distribution of total healthcare costs for patients with and without open tibial fractures

A univariate comparison of clinical and process -related variables for patients with and without an open fracture is shown in Table 3. In addition, total costs for patients with open and closed tibial fractures are visualized in Fig. 1.

**Table 3:** Comparison of clinical and process characteristics for open and *closed* tibial fractures (*n* = 358)

	Patients	Open (*n* = 51)	Closed (*n* = 307)	*p*
Clinical characteristics	Gender			0.079
	-Male	33 (64.7%)	158 (51.5%)	
	-Female	18 (35.3%)	149 (48.5%)	
	Age (years)	48 (29 – 60)	49 (37 – 63)	0.242
	ASA score			0.298
	-ASA 1	19 (37.3%)	139 (45.3%)	
	-ASA 2	23 (45.1%)	135 (44.0%)	
	-ASA 3	9 (17.6%)	33 (10.7%)	
	CVRF	26 (59.1%)	172 (58.9%)	0.981
	AO/OTA bone segment			<0.001***
	-41	6 (11.8%)	116 (37.8%)	
	-42	31 (60.8%)	109 (35.5%)	
	-43	14 (27.5%)	82 (26.7%)	
	AO/OTA fracture type			0.010**
	-A	22 (43.1%)	97 (31.6%)	
	-B	10 (19.6%)	129 (42.0%)	
	-C	19 (37.3%)	81 (26.4%)	
	AO/OTA fracture group			0.190
	-1	21 (41.2%)	141 (45.9%)	
	-2	12 (23.5%)	42 (13.7%)	
	-3	18 (35.3%)	124 (40.4%)	
	Antibiotic therapy			<0.001***
	-prophylactic	27 (52.9%)	286 (93.2%)	
	-therapeutic	24 (47.1%)	21 (6.8%)	
	Delayed-staged surgery	17 (33.3%)	27 (8.8%)	<0.001***
	Type of definite surgery			<0.001***
	-intramedullary nail	35 (68.6%)	105 (34.2%)	
	-plate osteosynthesis	14 (27.5%)	130 (42.3%)	
	-screw osteosynthesis	0	66 (21.5%)	
	-external fixator	2 (3.9%)	5 (1.6%)	
	-arthrodesis	0	7 (2.3%)	
	Superficial infection	0	0 (3.9%)	0.276
	Deep infection	7 (13.7%)	5 (1.6%)	<0.001***
	Nonunion	10 (19.6%)	8 (2.6%)	<0.001***
	Other complications	2 (3.9%)	6 (2.0%)	0.379
	Debridement	10 (19.6%)	9 (2.9%)	<0.001***
	Hardware removal	25 (49.0%)	96 (31.3%)	0.013*
	Re-osteosynthesis	8 (15.7%)	6 (2.0%)	<0.001***
	Other operations	10 (19.6%)	12 (3.9%)	<0.001***
	Mortality	1 (2.0%)	10 (3.3%)	0.619
Process characteristics	LOS to definite surgery (days)	0 (0 – 5)	1 (0 – 3)	0.950
	Total length of antibiotic therapy (days)	6 (4 – 11)	1 (1 – 2)	<0.001***
	Total LOS (days)	11 (7 – 25)	6 (4 – 13)	<0.001***
	Number of operations	2 (1 – 3)	1 (1 – 2)	<0.001***
	Number of hospital admissions	1 (1 – 2)	1 (1 – 2)	<0.015*
	Number of surgical day care admissions	0	0	0.883
	Number of ambulatory consults	7 (5 – 10)	5 (3 – 7)	<0.001***
Healthcare costs	Honoraria	€2,609 (1,484 – 4,013)	€1,494 (1,135 – 2,281)	<0.001***
	Materials (implants & screws)	€1,617 (1,070 – 2,501)	€1,002 (467 – 1,397)	<0.001***
	Hospitalization	€4,519 (2,876 – 10,271)	€2,465 (1,643 –5,341)	<0.001***
	Day care stay	€0 (0 – 177)	€0 (0 – 86)	0.588
	Pharmaceuticals	€1,448 (981 – 3294)	€978 (862 – 1,724)	<0.001***
	Total healthcare costs	€11,061 (7,135 – 19,883)	€6,632 (4,714 – 10,095)	<0.001***

Categorical variables are presented as numbers and percentages, continuous variables as median with interquartile distributions

*p < 0.05, **p ≤ 0.01, ***p < 0.001 (*p* < 0.05 is considered to be significant)

*Abbreviations: CVRF* cardiovascular risk factors, *LOS* length of stay

### Which clinical parameters drive the healthcare costs of open tibial fractures?

In order to determine the importance of the clinical variables for driving healthcare costs, a multivariate linear model was fitted using total healthcare costs as the dependent variable. The resulting model (all variables costing model - *AV-CM*) had an R^2^ of 0.98 and an adjusted R^2^ of 0.97. Only the variable total LOS was deemed significant (Table 4, model AV-CM).

**Table 4:** Model results (*n* = 51)

	Model	AV-CM		NSRV-CM		LS-M	
	Dependent variable	Total costs		Total costs		Length-of-stay	
	Variable	t-value	*p*	t-value	*p*	t-value	*p*
Clinical characteristics	Gender						
	-Male	.	.	.	.	.	.
	-Female	.	.	.	.	.	.
	Age	.	.	0.55	0.586	1.32	0.193
	ASA score						
	-ASA 1	.	.	.	.	.	.
	-ASA 2	.	.	.	.	.	.
	-ASA 3	.	.	0.92	0.362	.	.
	CVRF	.	.	2.09	0.043*	1.86	0.069
	AO/OTA classification						
	-bone segment 42 ^a^	.	.	2.00	0.052	1.84	0.073
	-fracture type (A, B, C)	.	.	.	.	.	.
	-fracture group 3 ^a^	.	.	0.77	0.446	0.89	0.380
	Open fractures	.	.	.	.	.	.
	Antibiotic therapy						
	-prophylactic	.	.	.	.	.	.
	-therapeutic	.	.	0.95	0.346	1.637	0.109
	Delayed-staged surgery	.	.	.	.	.	.
	Type of definite surgery						
	-intramedullary nail	.	.	.	.	.	.
	-plate osteosynthesis	.	.	.	.	.	.
	-screw osteosynthesis	.	.	.	.	.	.
	-external fixator	.	.	1.23	0.228	.	.
	-arthrodesis	.	.	.	.	.	.
	Superficial infection	.	.	.	.	.	.
	Deep infection	1.66	0.104	3.81	<0.001***	3.49	0.001***
	Nonunion	1.99	0.053	1.66	0.104	.	.
	Other complications	.	.	.	.	.	.
	Debridement	.	.	1.66	0.105	1.56	0.126
	Hardware removal	.	.	0.84	0.404	.	.
	Re-osteosynthesis	1.21	0.233	.	.	.	.
	Other Operations	.	.	.	.	.	.
	Mortality	.	.	.	.	.	.
Process characteristics	LOS to definite surgery	.	.	excluded	excluded	excluded	excluded
	Total length of antibiotic therapy	.	.	excluded	excluded	excluded	excluded
	Total LOS	30.56	<0.001***	excluded	excluded	excluded	excluded
	Number of operations	0.77	0.448	excluded	excluded	excluded	excluded
	Number of hospital admissions	1.18	0.246	excluded	excluded	excluded	excluded
	Number of surgical day care admissions	.	.	excluded	excluded	excluded	excluded
	Number of ambulatory consults	.	.	excluded	excluded	excluded	excluded
Model	R^2^		0.98		0.67		0.54
	Adj R^2^		0.97		0.58		0.46

*p < 0.05 **p < 0.01 ***p ≤ 0.001 (*p* < 0.05 is considered to be significant)

^a^ Categories which were not selected in the final models where dropped from this listing

*Abbreviations: AV-CM* all variables costing model, *NSRV-CM* non-stay-related costing model, *LS-M* length-of-stay model, *CVRF* cardiovascular risk factors, *LOS* length of stay

However, this high predictive value is mostly due to the fact that the LOS related variables are inherently correlated to healthcare costs due to Belgium’s financing system. This is because a part of the actual reimbursement method involves, among other factors, multiplying the LOS with a daycare price. As hospitalization costs comprise a large part of the total costs it is to be expected that the LOS related variables are significant when fitting these variables to the total healthcare cost per patient.

Therefore, all stay related variables were excluded in a second model (non-stay related variables costing model – *NSRV-CM*), again with total costs as the dependent variable. The variables denoted with *excluded* in Table 4 model NSRV-CM were excluded. As a consequence, the performance of this second model decreased significantly. The model had a R^2^ of 0.67 and an adjusted R^2^ of 0.58 (Table 4, model NSRV-CM) shows the significant variables in this multivariate model: deep infection and CVRF.

### Which clinical parameters drive the LOS?

Total LOS is the most important driver for healthcare costs (t = 30.56, AV-CM, Table 4). This brought up the question whether or not it is possible to identify the (non-stay related) variables that drive LOS. In subsequent analyses a multivariate linear model was built using LOS as a dependent variable and the non-stay related variables as independent variables. The model (LOS model – LS-M) had an R^2^ of 0.54 and an adjusted R^2^ of 0.46. The variable deep infection was found to be significant in the multivariate model (LS-M - Table 4).

### How are the total healthcare costs of open tibial fractures, total LOS, deep infection, CVRF, and AO/OTA fracture type related?

**Fig. 2:**
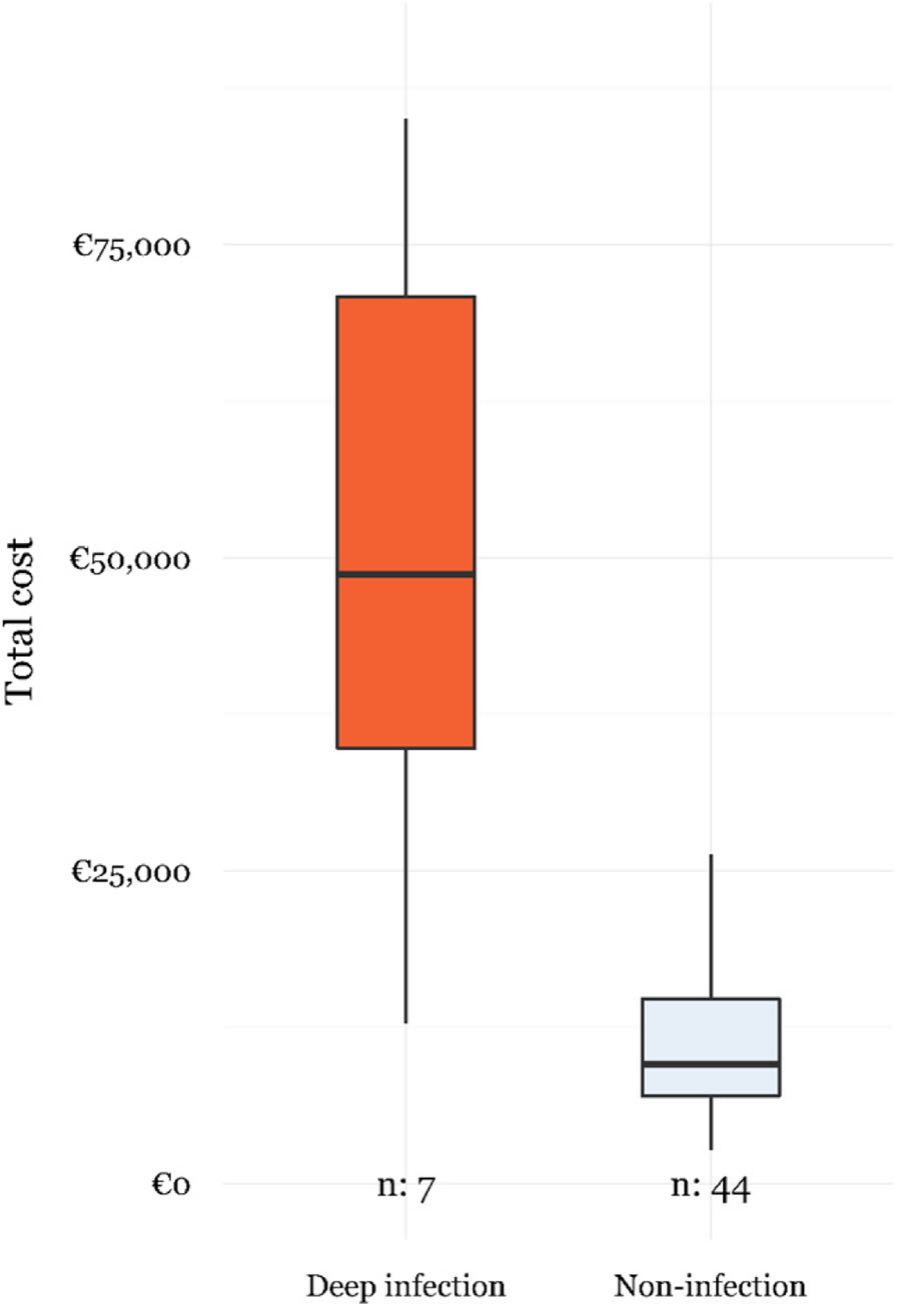
Distribution of total healthcare costs for patients with deep infection of open tibial fractures

Univariate analysis for open tibial fractures showed a 5-fold increase in the total healthcare costs (€48,702 [28,383-71,409] *vs.* €9,566 [6,781-15,094], *p*<0.001) (Fig. 2) and 6-fold increase of total LOS (60 [43-123] *vs.* 10 [7–18], p<0.001) in patients with a deep infection (*vs.* without deep infection).

The treatment costs and total LOS of open tibial fractures in patients with or without CRFV did not differ significantly (€13,321 [9,011-29,319] *vs.* €11,658 [6,992-13,804], *p* = 0.233) and 18 [9 – 34] *vs.* 11 [7 – 18], *p* = 0.138, respectively).

**Fig. 3:**
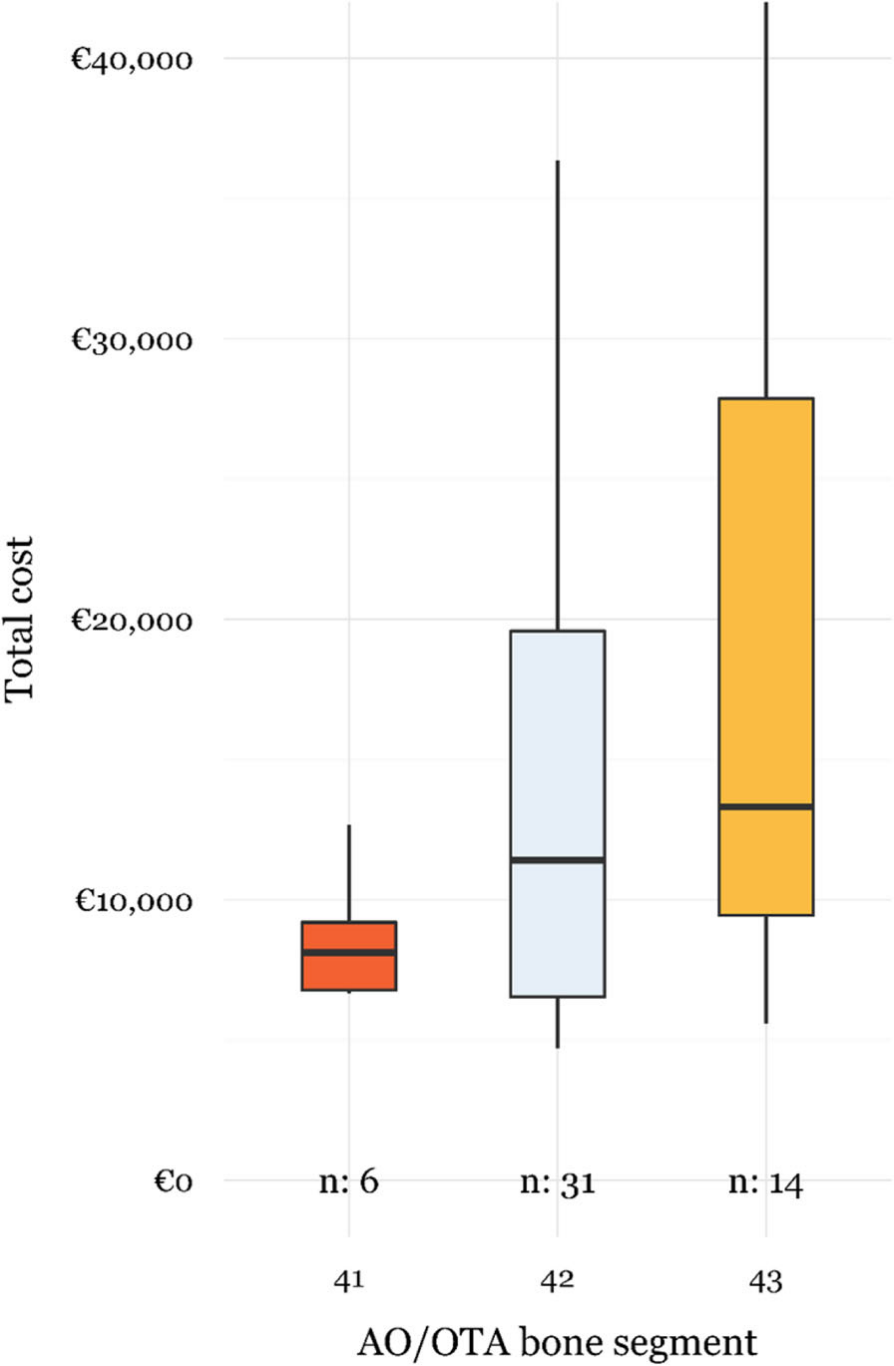
Distribution of total healthcare costs for patients with AO/OTA type 41, 42 and 43 open tibial fractures

Despite open AO/OTA type 42 tibial fractures were the most important bone segment fractures driving the total healthcare costs and total LOS, open AO/OTA type 43 tibial fractures turned out to be significantly more expensive (€13,304 [9,150-31,571] *vs.* €8,116 [5,669-10,084], *p* = 0.026) (Fig. 3) with significantly longer total LOS (20 [9 – 44] *vs.* 8 [4 – 11], *p* =0.028) compared to open AO/OTA type 41 tibial fractures.

### Do Gustilo type 3 open tibial fracture incur higher costs?

**Fig. 4:**
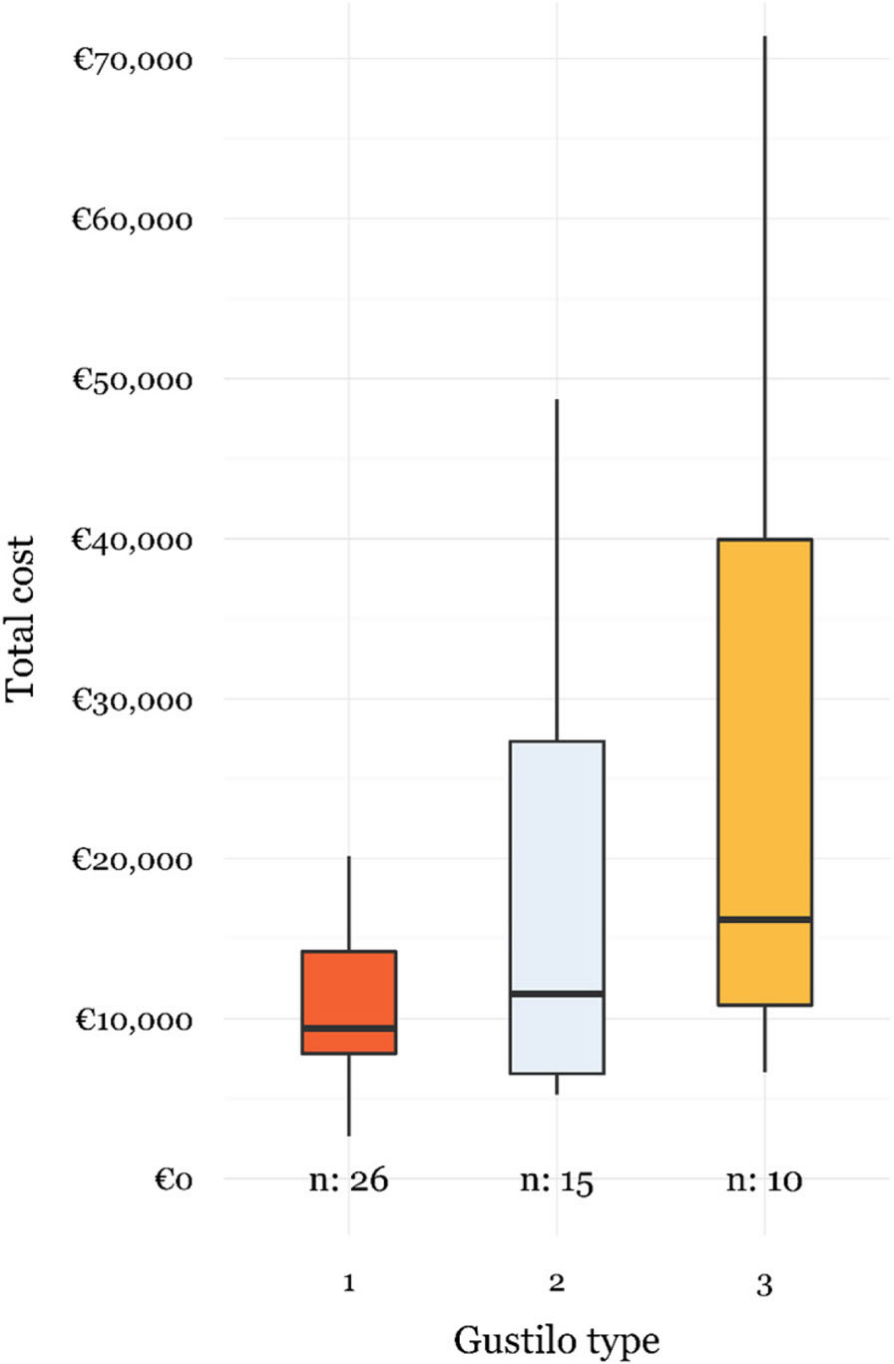
Distribution of total healthcare costs for patients with Gustilo type 1, 2 and 3 open tibial fractures

Univariate subanalysis for Gustilo type open tibial fractures showed significantly higher total healthcare costs (€16,163 [9,646 – 48,705] *vs.* €9,394 [7,157 – 14,802], p = 0.044) (Fig. 4), longer total LOS (26 [9 – 72] *vs.* 11 [7 – 17], *p* =0.044), as well as prolonged use of antibiotics (11 [6 – 24] *vs.* 5 [4 – 7], *p* =0.017) for type 3 compared to type 1 open tibial fractures. Subsequently, most deep infections were observed in patients with Gustilo type 3 open tibial fractures.

## Discussion

The goal of this study was to perform an exploratory analysis of treatment costs for open tibial fractures and define strategies in order to reduce these costs. A subset of clinical relevant variables was identified that drive the total healthcare costs and LOS. We hypothesized that Gustilo type 3 open tibial fracture patients incur higher costs.

Due to thin soft tissues layers covering the tibia shaft, open tibial fractures concerned here were predominantly AO/OTA type 42 fractures. These fractures were exclusively treated intramedullary. Open tibial fractures were approximately 8-times more frequently complicated by deep infection and nonunion in comparison with closed fractures. Subsequently, open tibial fractures required significantly more reinterventions (e.g. debridement, hardware removal, re-osteosynthesis). As a consequence, total healthcare costs of open tibial fractures were almost double as high compared to closed fractures. They mainly consisted of hospitalization costs. Total LOS was the most important variable driving the healthcare costs of open tibial fractures (t = 30.56). After exclusion of all process variables related to hospital stay, deep infection was identified as the most important clinical parameter driving the LOS (model LS-M) and total healthcare costs (model NSRV-CM) of open tibial fractures. In addition, CVRF were identified as significant driver of the total healthcare costs. Patients with open tibial fractures that were complicated by deep infection, showed a 6-fold increase in LOS and 5-fold increase in total healthcare costs. Despite the fact that open AO/OTA type 43 fractures were on average the most expensive tibial fractures, the multivariate analysis showed that the presence of an AO/OTA type 42 tibial fracture (in combination with other factors) was found to be a more important factor in driving the LOS and total healthcare costs. Gustilo type 3 open tibial fractures had a significantly higher total LOS and amount of healthcare costs, due to a higher rate of deep infection (30%).

Our results are in line with Page et al. [15], who demonstrated that patients with open tibial fractures have significantly increased total healthcare costs in the year following their injury, primarily due to a prolonged LOS and a greater number of ambulatory consults. Moreover, the fact that the total healthcare costs were to great extent determined by hospitalization costs, which in turn were driven by LOS, is in accordance with our previous observations in AO type 44B ankle fractures [3]. Unfortunately, whether or not the patient had an open ankle fracture, was not taken into account in this study. The median total healthcare costs and LOS for AO type 44B ankle fractures were €5,021 and 5 days, whereas for open tibial fractures this was €6,962 and 7 days, respectively. In contrast, the LOS and total healthcare costs of AO type 44B ankle fractures were strongly driven by the age and a delayed-staged surgery protocol. Antonova et al. [16] reported in 2013, higher median total healthcare costs for the treatment of AO type 42 fractures of $13,364 during a 2-year follow-up. In disregard of inflation, total costs in Euro are estimated at approximately €10.800 (index period 2006). Furthermore, the healthcare costs for tibial diaphyseal nonunion were twice as high here. Unfortunately, the total healthcare costs for open tibial diaphyseal fractures were also not specified in this study.

Although the median total cost per patient for the treatment of open tibial fractures was approximately twice as a high compared closed tibial fractures, total cost for the treatment of open tibial fractures was €955,026 *vs*. €2,758,172 for closed fractures. Since the total number of open tibial fractures was limited (14.3% of all fractures), the impact of strategies to curb the total costs of open tibia fractures (25.7% of the total cost) was also limited. However, Belgium’s hospitals are financed through a mixture of patient co-payments the health insurance system and the Ministry of Health [3], and therefore such strategies are certainly beneficial at the individual level. Another important limitation of these types of costing studies is the fact that it is often difficult to generalize the findings on an international scale. The difficulty in generalization stems from the fact that hospital financing is often very specific to a country. Currently no definitive way of internationally comparing costs for specific treatments exists. It might for example be the case - and it is in Belgium - that part of surplus funding for a given treatment is used to make up for inadequate funding of another type of treatment. Without understanding the intricate details of hospital financing in that specific country one might come to the erroneous conclusion that treatment y is cheaper compared to a country with sufficient funding for this treatment. (Time-driven) activity-based costing as proposed by Kaplan and Porter might bridge this gap, but requires investments in order to make treatment costs more transparent and easily measurable [17]. The University Hospitals Leuven is currently developing such a model and future publications could present a further refined cost specification for ankle fractures.

Compared to the literature, our deep infection rate (3.4%) was rather low [18]. Patients with open tibial fractures were significantly more affected by deep infection and nonunion. Gustilo type 3 open tibial fractures were significantly more affected. This is in accordance with Khatod et al. [19], who found that the Gustilo grading system of open fractures is a significant prognostic factor to indicate infectious complications. Moreover, the average time to surgery in their study was also not significantly different in infected versus non-infected cases.

It is also important to notice that, especially in patients with open tibial fractures, we observed a strong variance in terms of costs. We assume that most likely the total LOS is not only a result of surgical strategy, patient characteristics and surgeon’s decision, but is also a function of local guidelines, historically developed processes, habits and the individual preferences of the surgeon. Therefore, appropriate international consensus guidelines are required to improve not only the patient outcome but also reduce overall healthcare cost by focusing on reducing the LOS.

Deep infection was found to be the leading cause of the high total LOS and healthcare costs of open tibial fractures. Strict antibiotic guidelines and surgical protocols are adopted to minimize infection. Provided that the guidelines and protocols should be in the first place clinically beneficial, they may also serve as a tool to curb the total LOS and healthcare costs. Following the results of this study, we updated our treatment strategies for open fractures in order to improve patient outcome and reduce healthcare costs. First, current evidence states that long term systemic prophylactic antibiotic administration is not necessary [20]. Shortening this period will not only reduce healthcare costs but also reduce the change of creating bacterial resistance. Second, the time to definitive soft tissue coverage was shortened. A standardized protocol was developed with the colleagues from the department of plastic and reconstructive surgery to improve especially the logistic and planning of these procedures. Soft-tissue protocols consists of wound closure either with primarily or covered with adhesive drapes or VAC, preventing repeated debridement, and allowing rapid definite skin coverage. Finally, local prophylactic antibiotic strategies were improved. Current evidence indicates that locally delivered antibiotics or other antimicrobials could improve patient outcome [21–23]. Local delivery systems, like implant coatings, could be an asset in this field as well [24].

Please note, that the analysis presented here is based on an arguably limited sample of open tibial fractures, due to this a cautionary approach has to be taken when inferring properties about this sample and, in turn, about the population. This has been taken into consideration by limiting the number of variables in the multivariate model (see Methods) and by verifying the conclusion drawn below though bivariate analysis.

## Conclusion

In conclusion, total healthcare costs for open tibial fractures were approximately twice as high compared to closed fractures and mainly consisted of hospitalization costs, i.e. the cost of daily patient care. This study shows that total LOS was the most important variable driving total healthcare costs of open tibial fractures. Guidelines on appropriate LOS can help to improve efficiency, decrease variability, and curb the total healthcare costs. Open tibial fractures complicated by deep infection presented the most significant increase in LOS and in total healthcare costs. Besides that infection prevention is essential in high-quality care, it can also be an important contribution to make trauma care more cost efficient. To that end, patients with open tibial fractures must be treated according to strict antibiotic and surgical guidelines. In cases of deep infection, updated antibiotic protocols make it possible to administer intravenous antibiotics in outpatients (OPAT) with the same level of outcome and a shorter LOS. Multiple possible preventive measures like local antibiotics and implant coatings can be addressed. Finally, as has been evident, trauma research faces the problem of studies publishing heterogeneous small sized patient cohorts, therefore (multicentre) randomized clinical trials on infection prevention are mandatory to ultimately ensure the quality as well as standardization of trauma protocols and hospitalization processes (i.e. LOS).

## Abbreviations

AO/OTA: Arbeitsgemeinschaft fur Osteosynthesefragen/Orthopedic Trauma Association LOS length-of-stay
ASA-score: American Society of Anesthesiologists physical status score
AV-CM: All variables costing model
CVRF: cardiovascular risk factors
LS-M: LOS model
NSRV-CM: Non-stay related variables costing model

## Footnotes

1 LOO cross validation showed a somewhat decreased performance on unseen data which was deemed acceptable due to the limited number of available observations.
